# Indoor Air Quality Intervention in Schools: Effectiveness of a Portable HEPA Filter Deployment in Five Schools Impacted by Roadway and Aircraft Pollution Sources

**DOI:** 10.3390/atmos13101623

**Published:** 2022-10-05

**Authors:** Nancy Carmona, Seto Edmund, Timothy R. Gould, Everetta Rasyid, Jeffry H. Shirai, BJ Cummings, Lisa Hayward, Timothy V. Larson, Elena Austin

**Affiliations:** 1 Department of Environmental & Occupational Health Sciences, University of washington, Seattle, WA 98195, USA; 2 Department of Civil & Environmental Engineering, University of Washington, Seattle, WA 98195, USA

**Keywords:** indoor air quality, schools, portable air cleaners, aircraft pollution sources

## Abstract

The Healthy Air, Healthy Schools Study was established to better understand the impact of ultrafine particles (UFPs) on indoor air quality in communities surrounding Seattle-Tacoma (Sea-Tac) International Airport. The study team took multipollutant measurements of indoor and outdoor air pollution at five participating school locations to estimate infiltration indoors. The schools participating in this project were located within a 7-mile radius of Sea-Tac International Airport and within 0.5 mile of an active flight path. Based on experimental measures in an unoccupied classroom, infiltration rates of (a) UFPs of aircraft origin, (b) UFPs of traffic origin, and (c) wildfire smoke or other outdoor pollutants were characterized before and after the introduction of a portable high-efficiency particulate air (HEPA) filter intervention. The portable HEPA cleaners were an effective short-term intervention to improve the air quality in classroom environments, reducing the UFP count concentration from one-half to approximately one-tenth of that measured outside. This study is unique in focusing on UFPs in schools and demonstrating that UFPs measured in classroom spaces are primarily of outdoor origin. Although existing research suggests that reducing particulate matter in homes can significantly improve asthma outcomes, further investigation is necessary to establish the benefits to student health and academic performance of reducing UFP exposures in schools.

## 1. Introduction

Given that people spend 85% to 90% of their time indoors, the quality of indoor air is likely to have a significant impact on health, even though it is outdoor air that is regulated [1]. Increasing evidence has highlighted the health impacts of traffic-related outdoor air pollutants, including ultrafine particles (UFPs), on communities living in proximity to aircraft descent paths within the United States and internationally. The recently completed Mobile Observations of Ultrafine Particles (MOV-UP) study in King County, Washington, identified a clear, aircraft-associated footprint of UFPs under flight paths. Monitoring campaigns conducted in communities near airports in Seattle [2,3], Los Angeles [4–7], Atlanta [8], Boston [9], New York [10], and Amsterdam [11] have all identified elevated levels of total UFPs in proximity to international airports. This work has also highlighted differences in the pollutant mixtures between aircraft and roadway traffic sources [6,10,12,13], as well as differences in fuel-based emissions of UFPs from aircraft and roadway traffic sources [2,7].

Even though the spatial distribution of UFPs is still relatively unknown, minority and low socioeconomic status (SES) communities are often located closer to many UFP sources. A recent study in Boston, MA found that block group-level indicators of race/ethnicity and SES were related to the distribution of outdoor UFP concentrations [14]. Children are thought to be especially vulnerable to exposure to air pollution due to their higher ventilation rate and pulmonary surface area to body mass ratios, and relatively immature immune and respiratory systems [15,16]. A European review of UFP exposures in children suggests that the greatest predictors of high exposure in children were proximity to heavy traffic or proximity to cooking and cleaning activities [17]. School settings have been identified as priority environments for interventions to improve air quality, particularly in response to extreme events such as wildfires [18]. Portable air cleaners with HEPA filters may improve indoor air quality by removing particulates from the air [19]. Previous efforts to evaluate the impact of interventions to remove air pollutants in indoor spaces are limited and generally focused on residential environments [20,21].

The purpose of this proof-of-concept study is to assess the potential effectiveness of a portable HEPA intervention in reducing aircraft, traffic, and diesel-related exposures in highly impacted schools. We hypothesized that HEPA air cleaners would significantly reduce UFP and black carbon (BC) concentrations in classrooms and could provide solutions to reduce disparities in exposure within a metropolitan region. The present work is the first effort to examine the concentrations of indoor and outdoor UFP levels and the UFP removal efficiency of HEPA filters in schools located in airport communities.

## 2. Materials and Methods

### 2.1. Study Area and Study Design

Seattle-Tacoma International (Sea-Tac) Airport lies about 13 miles (~21 km) south of downtown Seattle, but several smaller cities such as SeaTac, Burien, Des Moines, and Normandy Park surround the airport. UFP levels have been found to be elevated near large airports and have different compositions and sizes than those from road traffic [22]. Puget Sound’s high population density makes this an important public health concern, particularly for sensitive populations such as children.

**Figure 1.**
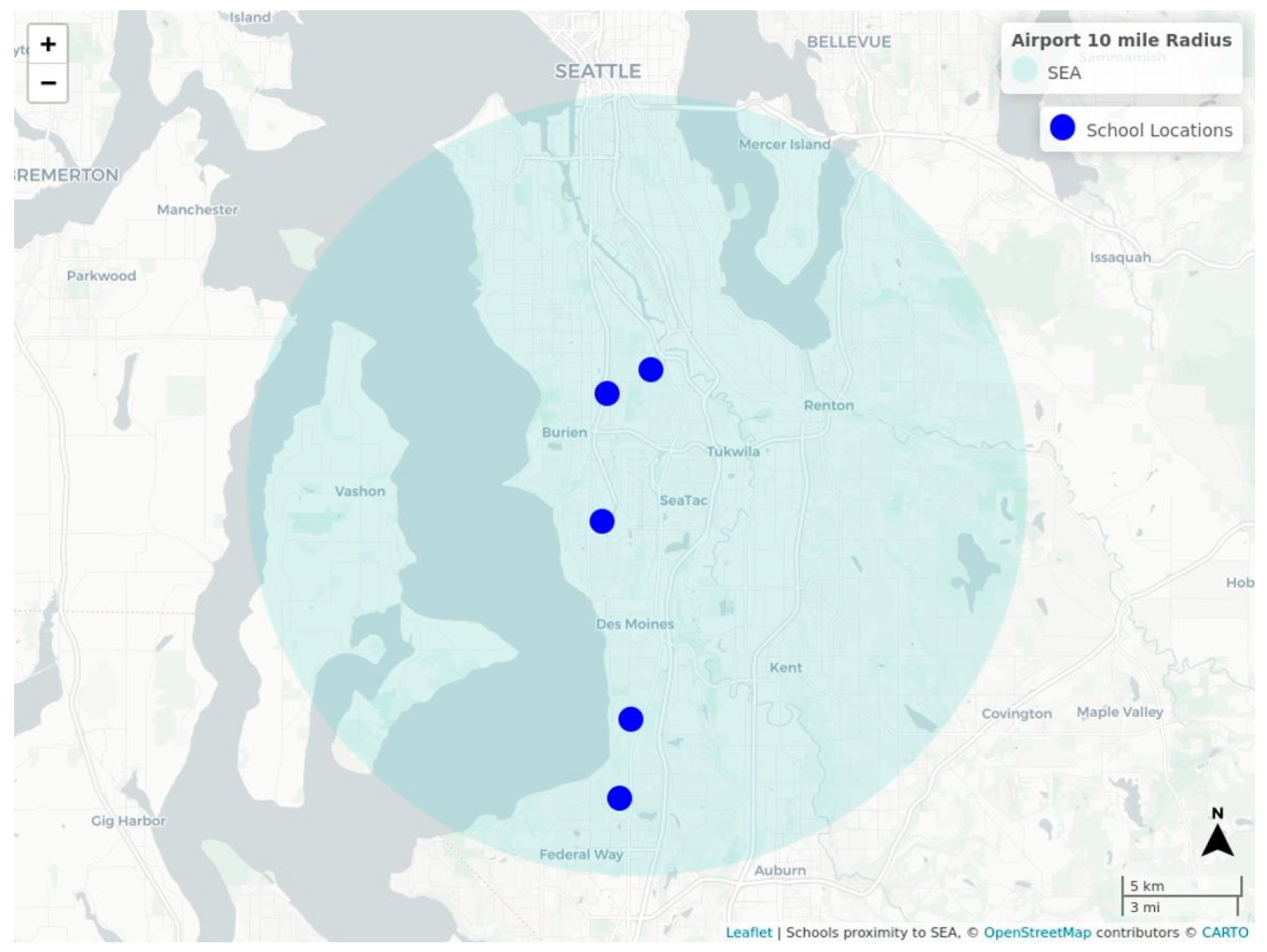
Map of school locations with an overlay of Sea-Tac Airport 10-mile radius. An interactive version of this map is available https://deohs.washington.edu/sites/default/files/mu/flights_near_schools.html (accessed on 28 September 2022). The interactive map also includes a layer illustrating the number of flights below 750 m in altitude and within one mile of schools in the year 2019). Map data were made available under the Open Database License: http://opendatacommons.org/licenses/odbl/1.0/. Any rights in individual contents of the database are licensed under the Database Contents License: http://opendatacommons.org/licenses/dbcl/1.0/.

Monitoring sites within the Federal Way and Highline School Districts were selected by the University of Washington research team with guidance from the Federal Way and Highline Public Schools partners. These school districts are particularly impacted by roadway and aircraft traffic. Five schools in the Sea-Tac Airport flight path were selected to evaluate the impact of airport traffic. Figure 1 shows the locations of participating schools with an overlay of Sea-Tac Airport 10-mile radius. The participating school buildings represent a variety of air handling designs and building ages. Infiltration of outdoor air pollution into classrooms was measured under normal operating conditions before and after deploying HEPA filters. Air monitoring took take place in spring and summer of 2021.

For each school, we computed the total count of 2019 flights that flew overhead within a one-mile radius of the school. The 2019 flight tracking data were used as a representative of normal conditions not impacted by COVID-19 pandemic flight reductions and were obtained from the Federal Aviation Administration through a Freedom of Information Request. School coordinates were obtained from the Washington Geospatial Open Data Portal from which the dataset contained all Washington State public schools for schools listed in 2021–2022 on the Washington State Office of Superintendent of Public Instruction (OSPI) School Directory. Data on flight counts per school were computed individually for the arrivals and departures of Sea-Tac Airport, Boeing Field (BFI), and Renton Municipal Airport (RNT) as well as the total overall flight counts from all three airports. Flights were counted if they were less than 750 m in altitude and within one mile of the school location. We performed the graphical representation of the data using the leaflet library in R and presented the flight counts as values of radius in logarithmic scale [23].

### 2.2. Portable Air Cleaner Selection

**Figure 2.**
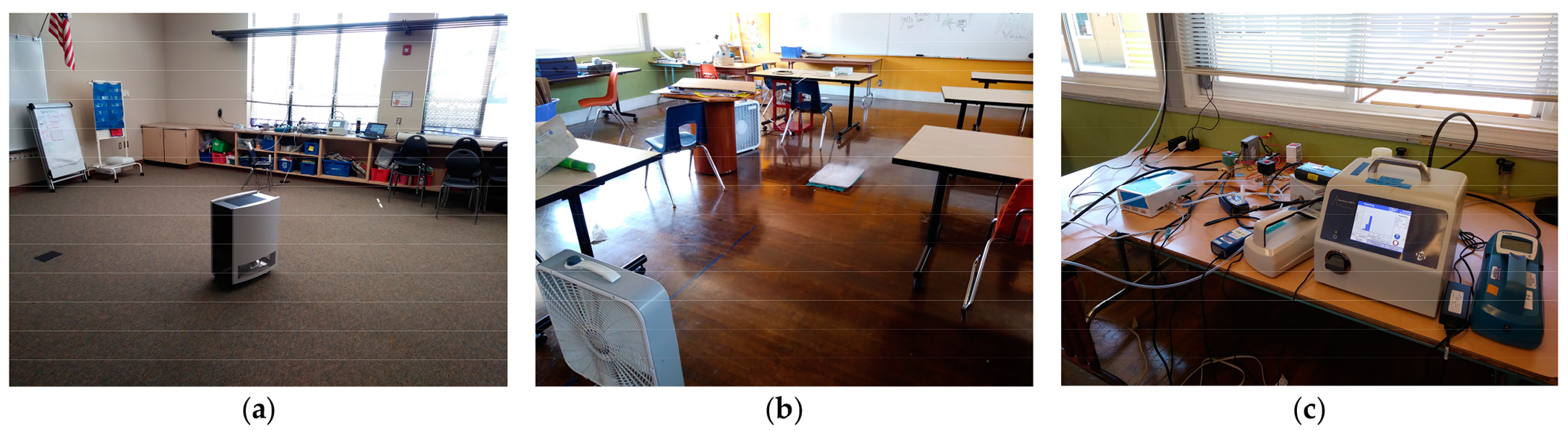
Instrument arrangement for indoor/outdoor air sampling, (**a**) Portable air cleaner placement in the center of unoccupied classroom. (**b**) Air exchange CO_2_ experiment. (**c**) Sampling instruments.

The portable air cleaner used in this study was the Blueair model 605 portable air cleaner with a HEPA-rated filter. Each device is evaluated by the Association of Home Appliance Manufacturers (AHAM) Institute to ensure it can provide a clean air delivery rate (CADR) for smoke and dust and supply adequate filtration for large spaces (~800 square feet). This device is expected to provide adequate filtration over a six-month period before a filter change is necessary and a HEPA filter with 99.9% efficiency at capturing ultrafine and fine particles, including those originating from wildfire smoke. The noise level is between 33 and 62 dB(A), depending on the fan speed setting. The portable air cleaner was placed in the center of the classroom away from walls and corners. The upright HEPA filter was placed on the floor of each classroom where its filtered air outlet was approximately 24 above the floor. Figure 2a shows the portable air cleaner placement in the center of an unoccupied classroom. The HEPA filter was set to “3” its highest flow rate at 500 cubic feet per minute (CFM).

### 2.3. School Site Selection

Sites were selected in consultation with school district partners to represent a range of building ages as well as proximity to flight paths and roadway traffic. All school sites selected were within 0.5 mile of an active flight path serving Sea-Tac Airport and within a 7-mile radius of the airport (see Figure 1). The classrooms where monitoring occurred were selected by school staff to be representative of the school or a particular part of the building in addition to being vacant of students at the time of our sampling. Characteristics of the monitored classrooms are shown in Table 1. Classroom ventilation varied by building, with some having central ventilation and other using room-specific ventilation units. All school classrooms, except School A 2nd floor, were located on the ground floor.

### 2.4. Outdoor Air Exchange Rate

The outdoor air exchange rate (AER) was measured in the first 2 to 3 h of each 48-h site visit. This measure of air exchange reflects the exchange of air between the indoor and outdoor space and is a component of total air exchange rate that also includes recirculation through the HVAC filter system. Since our primary interest was in the movement of outdoor air into indoor spaces, we focused on the measurement of outdoor AER in this project. We followed the protocol developed by the Harvard University T.H. Chan School of Public Health’s Healthy Buildings Program [24]. Since we were able to conduct our measurements in unoccupied buildings, we used the CO_2_ decay method to determine the air exchange rate from among the options presented in the Harvard Healthy Buildings Program guide. The Harvard Healthy Buildings Program method involves elevating the CO_2_ concentration in the test classroom and then measuring the declining CO_2_ concentration over time to enable determination of the decay rate. Dry ice was used to elevate the inside concentration of CO_2_. A tray was filled with dry ice and two box fans operated in the room to thoroughly mix the CO_2_ as the concentration increased. With CO_2_ elevated to four times or more the background level, the dry ice was removed from the room and the mixing fans shut off to begin the decay of CO_2_ concentration while the field technician exited the classroom. Figure 2b shows the air exchange experimental setup in a classroom.

Two CO_2_ analyzers were used to characterize CO_2_ concentration, one inlet near the center of the room and the other close to the windows along an outside wall of the classroom. Uniformity of CO_2_ concentration within the room was tracked over time, and with equivalent or very similar levels determined from the two monitors, we could then average the concurrent results as being representative for the entire room. The rate of decay without CO_2_ sources in the room is based on air exchange from (1) infiltration of air from the outside, and (2) the active ventilation system in the building. An adequate time series of CO_2_ decay is attained once the concentration drops to about one-third of the starting elevated level. The CO_2_ data from the time at which sources are removed and the decline begins, through the time at which ventilation characteristics are altered by opening doors or people reentering the room, define the decay rate of CO_2_, used to determine the air exchange rate. The measured in-room CO_2_ minus the ambient outdoor CO_2_ concentration is the quantity of interest to use in determining the air exchange rate. We used a dynamic mass balance model (Equation (1)) to calculate the outdoor exchange rate:

(1) $${\left(C_{classroom}\right)}_{t}=C_{indoor_{background}}+A_{0}*\text{exp}(-\text{k}*\Delta \text{t})$$

where $C_{classroom}$ is the concentration within the classroom at time *t*; $C_{indoor_{background}}$ is the initial indoor concentration; *k* is the deposition rate; and Δ*t* is the study sample period. *A*_0_ is defined as $C_{peak}-C_{indoor_{background}}$. The dynamic model accounts for CO_2_ moving in and out of an indoor microenvironment. The model assumed that there were no indoor sources, perfect mixing and no mass loss or gain due to differences in gas-phase concentrations or temperature and relative humidity conditions between indoors and outdoors [24].

### 2.5. Air Quality Sampling and Analysis Methods

Indoor and outdoor concentrations of selected air pollutants were measured over two consecutive 24-h time intervals concurrent with the outdoor air exchange rate measurements. The air pollutant measurements conducted for this pilot scale study were designed to accomplish three inter-related objectives: (1) determine the outdoor air exchange rate, (2) characterize the indoor pollutant concentrations and the outdoor ambient air pollutant concentrations and (3) assess the effectiveness of installing a portable air cleaner in the test classroom. The instruments used to measure the pollutants of interest are presented in Table 2. The UFP instruments provide a number count concentration, not a mass concentration measurement. The BC devices use a light absorption method to estimate the mass concentration of BC particles captured on an internal filter material. Figure 2c shows a picture of the instruments arranged in a classroom.

Classrooms were assessed twice with this research-grade sampling methodology. For most visits, a solenoid timer valve was set to alternate 5-min indoor and outdoor measurements with results stored with a 10-s time resolution. We trimmed the first two minutes of each 5-min NanoScan sample to account for potential mixing of indoor and outdoor air within the same one-minute scan, following the switch of the valve position between the two inlet locations.

**Figure 3.**
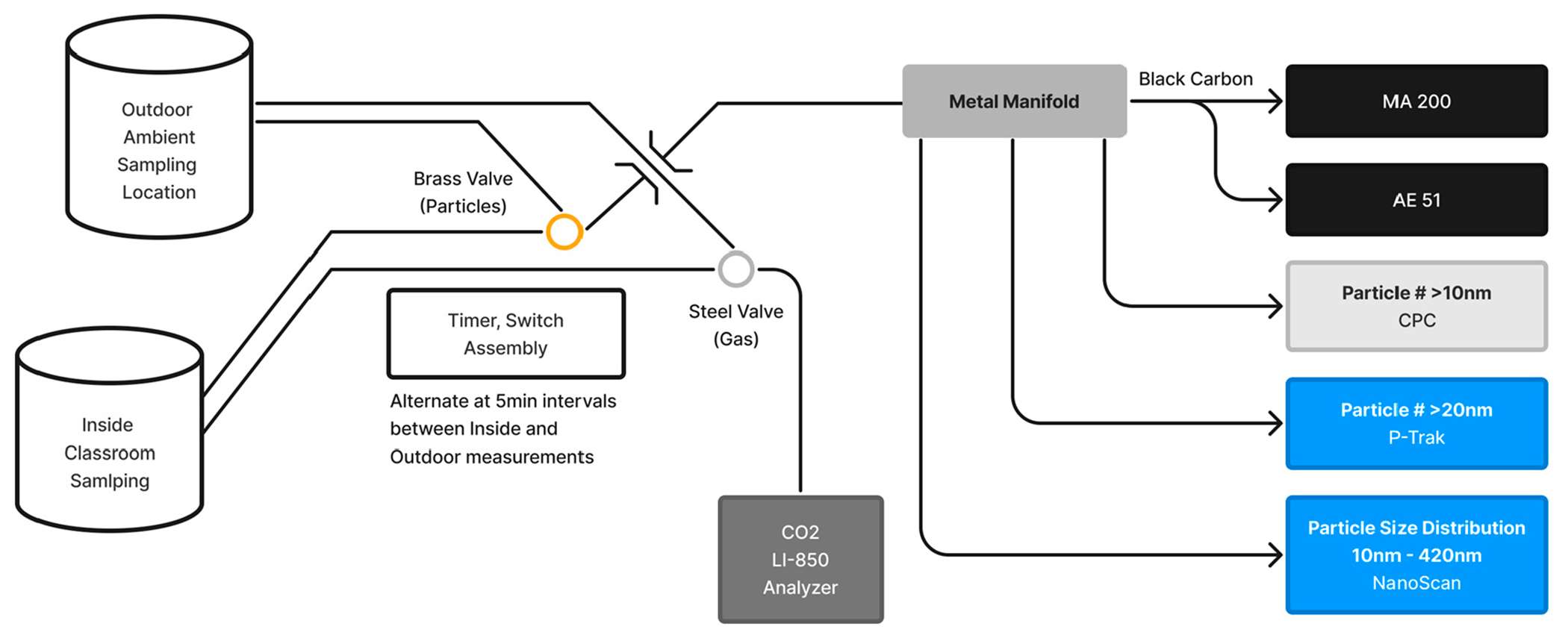
Pneumatic configuration of sampling instruments.

An inlet line to sample ambient air outside the classroom was installed using a slightly open window that was then backfilled with shim material and sealed with duct tape, or by use of an available conduit to the outside from within the classroom. At the first three classroom deployments, separate instruments were used for the indoor and outdoor air sampling, but from the fourth site visit starting in June 2021, a timer and valve switch mechanism was used to alternate the inlet to the monitoring instruments between an indoor and outdoor location every 5 min (e.g., timer switched the valve at hh:00:00, hh:05:00, hh:10:00, etc.). Figure 3 illustrates the configuration of instruments for indoor and outdoor air sampling using the switch valve.

### 2.6. Estimating Average Infiltration

Each school site was visited on two occasions over the measurement period of this project. At each visit, indoor and outdoor air quality was measured for 24 h prior to a portable HEPA intervention and 24 h after a HEPA filter intervention. These data provided the basis for estimating the average infiltration rate of particles into the indoor space. Average infiltration (see Equation (2)) was calculated from 30-min averages of indoor and outdoor count concentrations and the ratio was defined as infiltration. This required an assumption that the pollutants measured indoors were attributable to outdoor sources [25].


(2) $$\text{Infiltration}=\frac{{\text{Pollutant}}_{\text{indoor}}}{{\text{Pollutant}}_{\text{outdoor}}}$$

Removal effectiveness for the HEPA filter was calculated according to Equation (3) across the mean observations of our study [26].


(3) $$\text{Effectiveness}=1-\frac{{\text{Infiltration}}_{\text{HEPA}}}{{\text{Infiltration}}_{\text{noHEPA}}}$$

### 2.7. Statistical Anlaysis

We also estimated the percent removal of the HEPA cleaner using a regression approach. A log-log multivariate linear model regressed the indoor concentration of particles to a 30-min outdoor lagged concentration (see Equation (4)).


(4) $${\text{log}(\text{Pollutant}}_{\text{indoor}})=\text{log}\left({\text{Pollutant}}_{\text{lagged outdoor}}\right)+\text{HEPA}+\text{School}$$

A 30-min lag was selected based on time series data indicating a lag between outdoor peaks and subsequent indoor peaks. A school-specific adjustment was used to account for differences between schools, and a term indicating the presence of HEPA filter or not was included. Based on the coefficient estimated for the HEPA term, the removal effectiveness for the HEPA filter was calculated (according to Equation (3) above) across the mean observations of our study. The log-log model (see Equation (4)) was used to predict the concentration of particles in indoor air when outdoor concentrations were assumed to be 5000 particles/cc which was the median in our dataset. Confidence intervals were generated based on propagating the error terms from the regression output.

We conducted a two-sample Wilcoxon Rank Sum test to determine if the infiltration before the HEPA filter intervention was significantly higher than after the intervention (*p* < 0.5). We also calculated Pearson’s Correlation Coefficient to better understand the relationship between infiltration with and without the HEPA filter.

All analyses were conducted in R version 4.1.1. Packages used for analysis and output of results included data.table [27], ggplot2 [28], emmeans [29], zoo [30], psych [31], GPArotation [32] and dplyr [33].

## 3. Results

The dates of sampling in the Federal Way and Highline schools are presented in Table 3, along with the flight direction of aircraft at Sea-Tac Airport relative to the school location.

Over the course of these deployments, 10-s data were collected both inside and outside the school using the instruments and measurement protocols described in the Methods section. This allowed for detailed information on CO_2_, BC, and particle size to be characterized. The TSI NanoScan instrument occasionally would develop operating errors over the course of the sampling. Table 4 presents a summary of the percentage of time the NanoScan instrument produced errors during the school deployments. For time periods when the NanoScan data were not available, the TSI condensation particle counter (CPC) instrument measurements were substituted for the total concentration of particles (CPC does not measure multiple size ranges like the NanoScan). CPC data were not used to determine pollutant source.

Outdoor exchange rates were calculated using the CO_2_ decay method described above. Overall, the outdoor air exchange rates ranged from 0.6/h to 4.4/h, highlighting the variability in direct exchange of air with the outdoors at the different school sites (Table 5).

### 3.1. Outdoor Concentration

**Figure 4.**
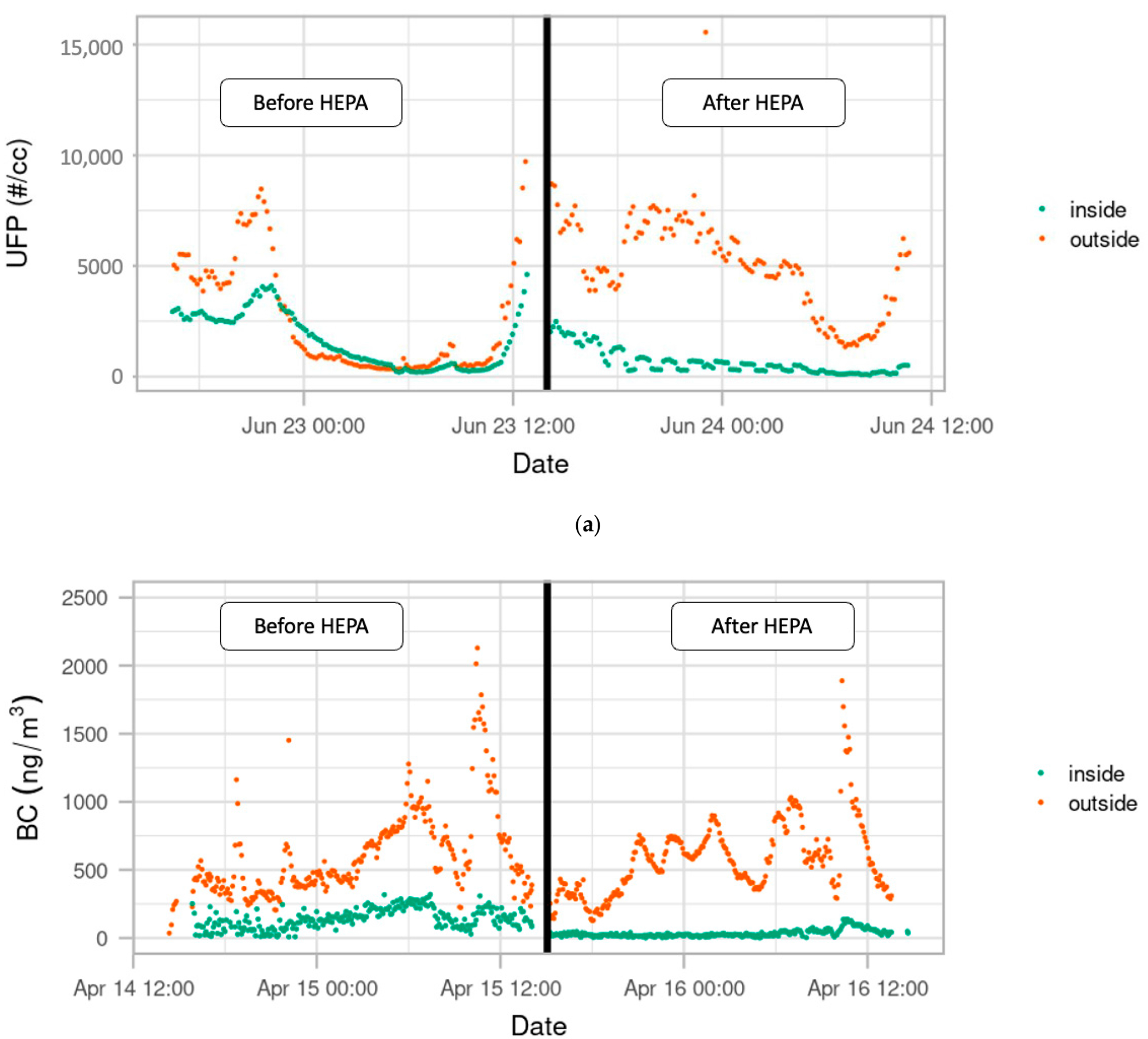
Indoor and Outdoor concentration of UFP Count and Black Carbon Particles at example schools. (**a**) Indoor and Outdoor concentration of total particle count before and after portable HEPA filter deployment. (**b**) Indoor and Outdoor concentration of Black Carbon before and after portable filter deployment.

The outdoor concentration observed at each of the five schools represents only four days of non-concurrent sampling. It is therefore difficult to directly compare the concentration of particles across the locations. Although there were distinct differences in total pollutant concentration at the different sites, these differences are likely not representative of the year-round average differences at these sites. However, the indoor and outdoor monitoring allowed for the comparison of the infiltration dynamics over time (Figure 4).

### 3.2. Observed Impact of HEPA Filter

The impact of the HEPA filter was evaluated by analyzing the relationship between the indoor and outdoor concentrations of pollutants measured over the course of deployment. In Figure 4, visual inspection suggests an effect from the use of the portable HEPA filter for both UFP and BC. The ratio of indoor-to-outdoor air pollution was calculated for each pollutant in order to assess the impact of the HEPA filter. The HEPA filter removed many of the pollutants that caused a spike due to infiltration when the HEPA filter was not present. Figure S1 (Supplementary Information) shows the change in the indoor-to-outdoor ratio of UFP measured at each visit, before and after she portable HEPA filter deployment.

Combining the data across all school locations, we found a significant reduction in pollutants after the HEPA filter deployment. Table 6 presents the estimated infiltration rates with and without portable HEPA filter deployment as well as the associated confidence intervals for all estimated values.

The total particle number (general traffic), particles of aircraft origin (d = 15.4 nm), and BC all decreased substantially after the HEPA filter deployment. Before the HEPA filter deployment, approximately half of all outdoor particles were measured indoors. After the HEPA filter deployment, approximately 1/10th of all outdoor UFP were measured indoors. The removal of outdoor particles infiltrating into the indoor space attributed to the portable HEPA filter is estimated to be 83% removal for UFP, 67% removal for aircraft, particles, and 73% removal for heavy-duty traffic particles. This represents a removal percent of 83% for particles of outdoor origin (Equation (3)). The estimated median removal indoors is moderately significant among particle types, suggesting that the HEPA filter intervention is effective for all outdoor particle air pollutants, including those of aircraft, wildfire, and roadway origin.

**Figure 5.**
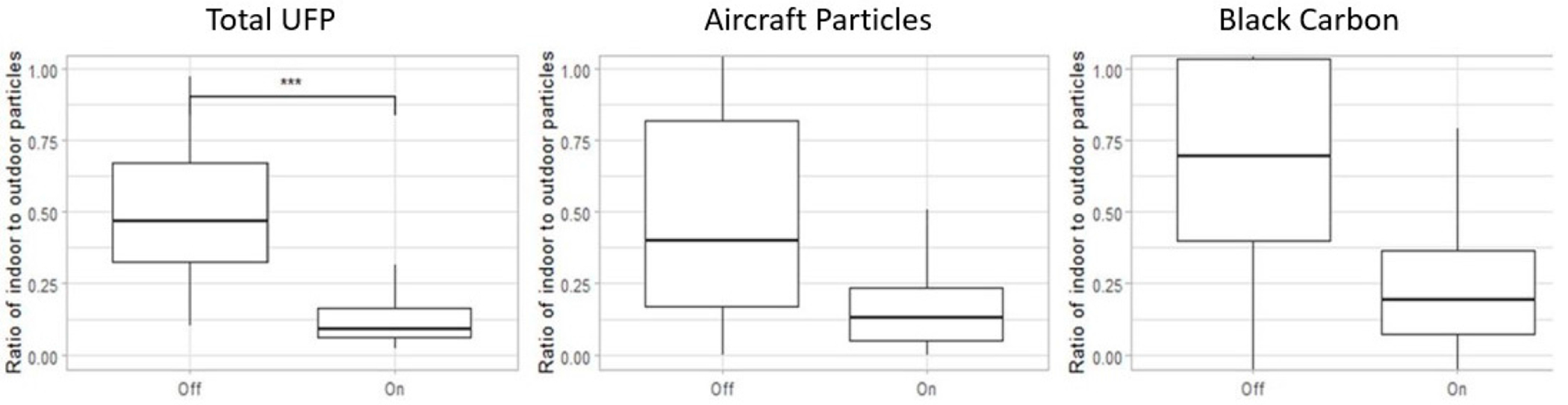
Infiltration ratio of different particle types before and after the portable HEPA filter intervention. This data represents the range of indoor/outdoor ratio of pollutants across all five schools. In the boxplote, the upper whisker represents the maximum, the top of the box represents the 75th percentile, the middle line represents the median or 50th percentile, the bottom of the box presents the 25th percentile, and the lower whisker represents the minimum. A two-sample Wilcoxon Rank Sum oest confirms that the infiltration before the HEPA filter intervention is significantly higher than after the intervention (*p* < 0.5), for each of the three particle sources. *** indicates the results are statistically significant.

We conducted a two-sample Wilcoxon Rank Sum test to better understand the relationship betweee infiltration and the HEPA filter intervention. A two-sample Wilcoxon Rank Sum test confirmed that the infiltration before the HEPA filter intervention was significantly higher than after the intervention (*p* < 0.5), for each of the three particle sources (Figure 5).

We also calculated Pearson’s Correlation Coefficient to better understand the relationship between infiltration with and without the HEPA filter. We found that, without the HEPA filter, there was a 40% correlation (moderate) between the indoor and outdoor measures. When the HEPA filter was deployed, there was a 9% correlation (weak) between the indoor and outdoor measures. We also found that this relationship between indoor and outdoor air quality persisted for up to 60 min after the HEPA filter was turned off, but that there was no observable correlation between indoor and outdoor when the HEPA filter was deployed. This can be observed in Figure 4 where the indoor concentration closely follows the change in outdoor concentration before the introduction of the HEPA filter (moderate correlation). After the introduction of the HEPA filter, there is no obvious relationship between the change in outdoor concentration and the change in indoor concentration.

### 3.3. Modeled Impact of HEPA Filter

**Figure 6.**
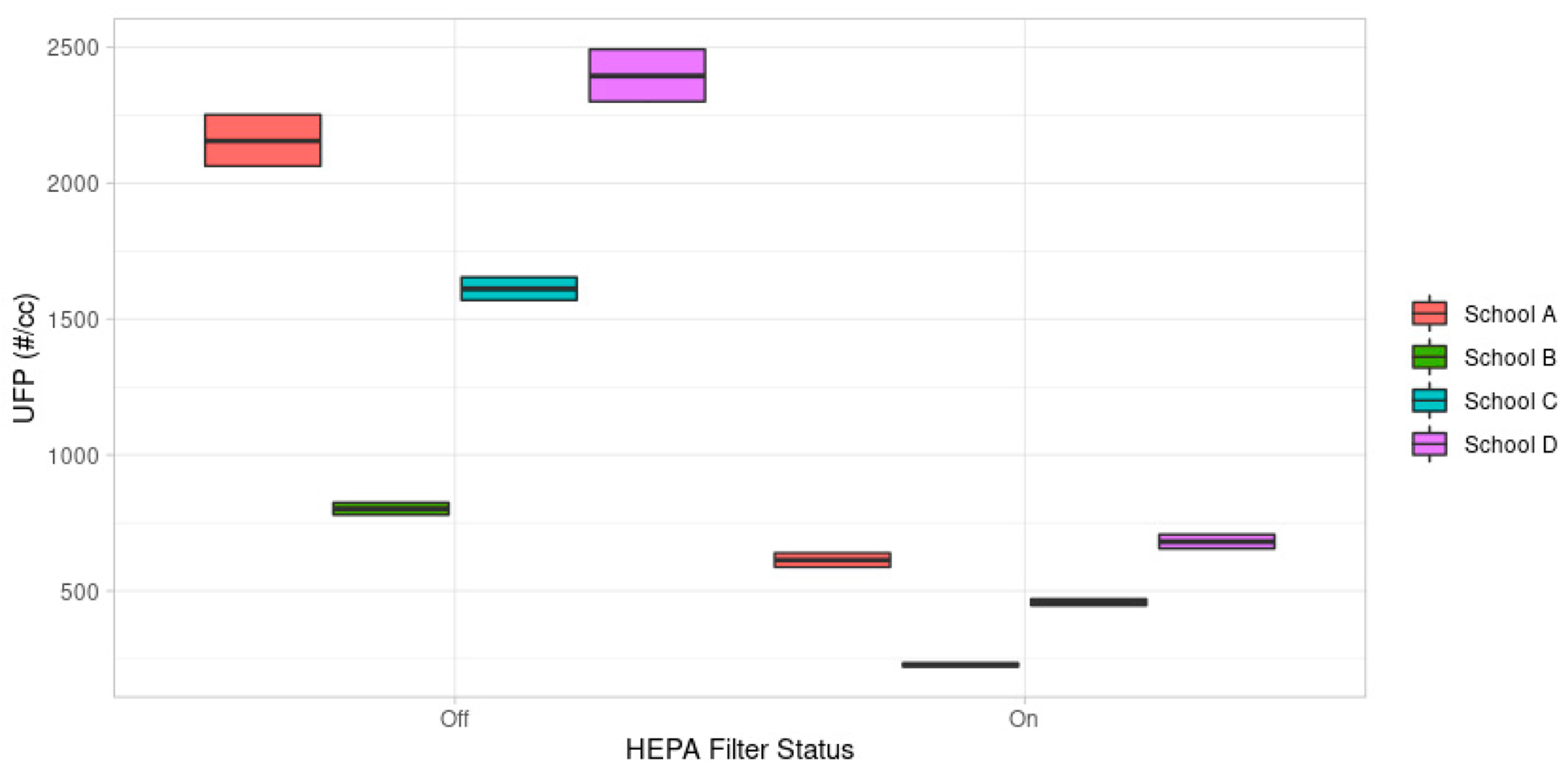
Prediction of indoor concentrations with and without a portable HEPA filter deployed in the classroom. The middle bar represents the mean, and the top and bottom bars represent the upper and lower 95% confidence interval. We assumed an outdoor concentration of 5000 #/cc to predict the indoor concentrations. School E was not included in this model due to multiple 0 values for the indoor air quality measurement.

In order to better understand the overall impact of HEPA filtration, we developed a regression model to predict indoor concentration based on the school location, use of a HEPA filter, and average outdoor concentration over the previous 30 min (Equation (4)). This model assumed that the indoor concentration represented a fraction of the outdoor concentration (log-log model). We then predicted the average indoor air quality concentration at each school, for a fixed outdoor concentration of 5000 #/cc with and without the HEPA filter intervention. We saw a statistically significant decrease in indoor air quality in all the schools, with School A having the highest infiltration rates with and without the HEPA filter intervention (Figure 6). School E was not included in the model as there were multiple indoor concentration values of zero observed after the HEPA filter deployment, making it impossible to include this location in the log-log model.

Overall, we estimated that the HEPA filter effectiveness was 71% [95% CI: 70–72%] across the measurement conditions, after accounting for school-specific differences. This regression result is consistent with the result observed when calculating HEPA filter effectiveness using the ratio of indoor-to-outdoor pollutants (Figure 6). We found that the prevention of infiltration varied between schools. In the next phase this model will be further expanded to include information on building age and ventilation type.

### 3.4. Overall Distribution of Pollutants

**Figure 7.**
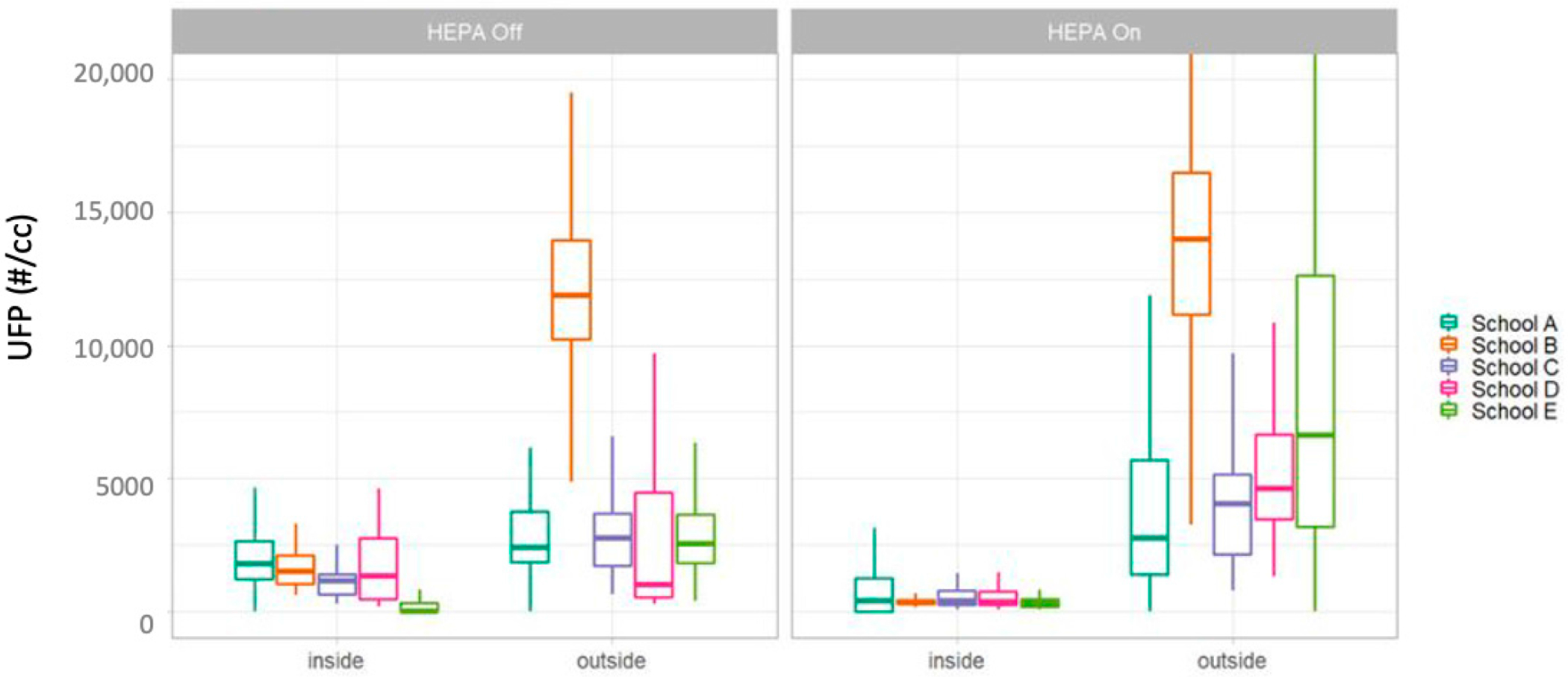
Distribution of UFPs before and after HEPA filter deployment at all five school locations. In the boxplots, She upper whisker represents the maximum; the top of the box represents the 75th percentile; the middle line represents the median or 50th percentile; the bottom of the box presents the 25th percentile; and the lower whisker represents the minimum.

**Figure 8.**
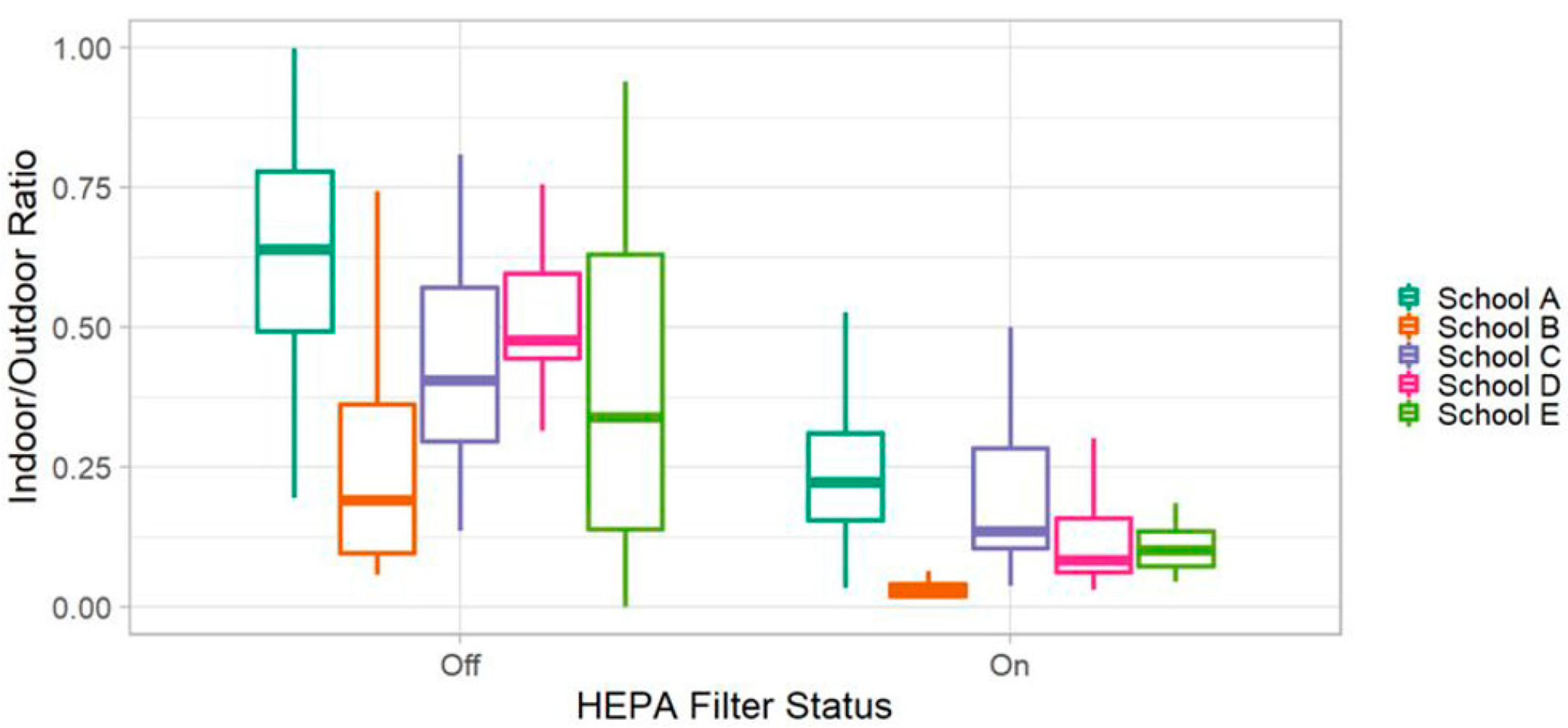
Distribution of indoor/outdoor ratio values for UFPs at each school location. Values were computed on the 30-min timescale. In the boxplote, the upper whisker represents the maximum, the top of the box represents the 75th percentile, the middle line represents the median or 50th percentile the bottom of the box presents the 25th percentile, and the lower whisker represents the minimum.

We found that prior to HEPA filter deployment, outdoor concentrations of UFPs, ultra-UFs, and BC are substantially higher than those measured indoors. This is consistent for all measured particulate pollutants. We consistently find that the total indoor concentrations are lower after the HEPA filter deployment, as shown below. Consistent with the findings of this paper that the portable HEPA filter has a significant impact on indoor air quality, we observed a reduction in the pollution from outdoor sources persisting in the classroom environment (Figure 7). The indoor/outdoor ratio also varied by school location. In Figure 8, we present the results of the observed 30-min indoor/outdoor ratios at each school location. Consistently, there are lower ratio after the HEPA filter deployment, but the magnitude of this change varies by school.

### 3.5. Spatial Distribution of Flightpaths

This study team was particularly interested in determining the potential removal of UFPs in airport communities. King County schools had a median of 154.5 nearby flights, with 102.5 nearby arrivals and 35 nearby departures (Table 7). Nearby flights were defined as flights below 750 m and within a one-mile radius of a school.

Schools participating in the present study had significantly more nearby flights, nearly all of them associated with Sea-Tac Airport. Participating schools had a median of 61,240 nearby arrivals and 53,547 nearby departures in 2019 (Table 8). We found that the schools participating in the study were substantially impacted by aircraft emissions from overhead flights when compared to the average school in King County.

**Figure 9.**
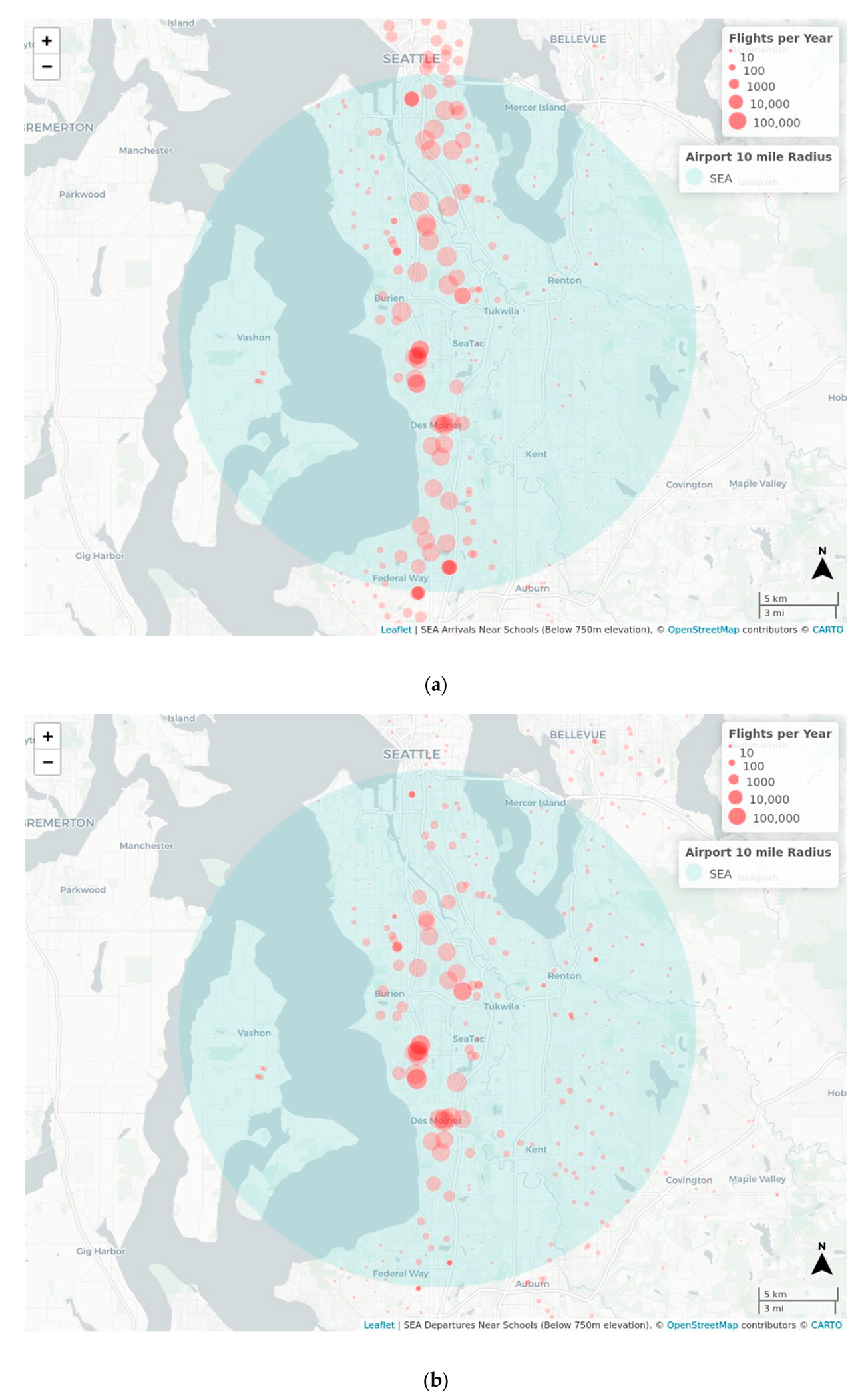
Number of flights in 2019 below 750 m and within a one-mile radius of a school, (**a**) Number of Sea-Tac Airport arrivals with overlay of the 10-mile radius for Sea-Tac Airport, (**b**) Number of Sea-Tac airport departures with overlay of the 10-mile radius for Sea-Tac Airport. An interactive version of this map can be found at https://deohs.washington.edu/sites/default/files/mu/flights_near_schools.html (accessed on 28 September 2022). Map data were made available under the Open Database License: http://opendatacommons.org/licenses/odbl/1.0/. Any rights in individual contents of the database are licensed under the Database Contents License: http://opendatacommons.org/licenses/dbcl/1.0/.

Figure 9 illustrates the number of arrivals and departures from Sea-Tac Airport and regional airports. The spatial distribution of departures is more closely centered south of SeaTac while arrivals extend north of SeaTac.

## 4. Discussion

This pilot study aimed to determine whether HEPA units are a feasible intervention to reduce aircraft UFP exposures in a school setting. Measurements in the present study were only conducted over a total of four days in each classroom, it would be informative to continue longer-term monitoring to better understand the impact of UFPs across this area. UFPs are not routinely measured in the outdoor environment by air quality agencies across the United States. Because of the lack of health-based regulatory standards as well as limited long-term monitoring data, it is difficult to compare the magnitude of the outdoor concentrations observed in this study to typical outdoor concentrations.

However, in recent years, there have been special studies in Pittsburgh, the Netherlands, New York, Montreal, Seattle, and Los Angeles that confirm UFPs are elevated near roadways, near industrial sites, in urban cores, and in proximity to flight paths [2,34–36]. There are strong gradients of exposure to UFPs observed in these studies, with UFPs decreasing to background levels within 100 m of sources.

To inventory available regulatory UFP monitoring data, we searched the EPA Air Quality System (AQS) database and contacted select local air quality agencies across the US. We found some form of UFP monitoring data near Baltimore, Miami, New York, Saint Paul, Pittsburgh, Los Angeles, and Seattle. In general, these special studies were either designed as short-term mobile monitoring studies or snapshot designs, where monitors were rotated among fixed sites for a year or less [2,37–40]. The New York State Department of Environmental Conservation (DEC) collected one-minute UFP count data across seven sites in New York State over the year 2017 at near-road, urban, suburban, and state park locations. The dataset clearly demonstrates UFP gradients away from roadways, with a site located directly next to a freeway in Queens, NY, reporting 1.5 to 2 times greater concentrations of UFPs than a site located only 300 m downwind of the road.

A study in Boston, MA, looked at the infiltration of aircraft related UFP into residential buildings in proximity to flight paths [5]. Hudda et al. found that median outdoor concentrations of UFP were 19,000 #/cc when the residence was downwind of the flight path and 10,000 #/cc during other wind conditions. The authors also found significant infiltration of aircraft particles into local residences and calculated a 33% decrease in indoor concentration after a portable HEPA filter was installed. A decrease in aircraft particles is consistent with the findings of this study, although HEPA filter effectiveness and infiltration rates were not calculated in the Boston project.

### 4.1. UFP Infiltration

Few studies have examined UFP levels in the classroom environment. Unlike our present study which measured UFPs in an unoccupied classroom, Mullen et al. measured UFPs during normal occupancy. Mullen et al. measured particle number (PN) concentrations inside and outside six classrooms in northern California and found that exposures appeared to be primarily attributed to outdoor sources [41]. Weichenthal et al. characterized UFP counts in 37 occupied classrooms in rural Ontario during winter and developed a model to predict exposures based on ambient weather conditions, classroom characteristics, and outdoor UFPs [42]. The study found that windspeed and outdoor UFPs were important determinants of classroom UFP levels. Weichenthal et al. found that predictive models based on outdoor UFP data perform reasonably well in estimating classroom UFP counts when indoor UFP sources were not present [42].

There are also a limited number of studies examining the infiltration of outdoor particles into the indoor environment of schools. Infiltration of outdoor particles into the indoor environment has been assessed in Barcelona schools. Rivas et al. assessed infiltration of traffic related emissions including UFPs and found that the median indoor/outdoor ratio ≤ 1 indicating that the outdoor traffic related sources contributed to indoor concentrations [43]. Infiltration factors have also been found to be different based on sources, with traffic components having indoor/outdoor ratios of 0.31–0.75 in the cold season and 0.50–0.92 in the warm season. However, building age and window material were not found to be a major determinant of indoor pollutant concentrations.

### 4.2. HEPA Filtration Intervention

In the Healthy Air, Healthy Schools Phase 1 project, we estimated that HEPA filtration resulted in 70% to 80% lower UFPs as compared to no additional filtration. This result is consistent with the findings from Boston. Recent controlled interventions have established improvements in symptoms of children with asthma after a HEPA filter intervention in their homes [44–47]. These studies also show consistent improvement in the indoor air quality of these homes after HEPA filter intervention. However, none of these studies directly evaluated UFPs, the primary pollutant of interest in this study, instead focusing on the PM_2.5_ fraction of air pollution.

Studies evaluating the impact of HEPA filtration in school settings are limited. Nine studies were identified that assessed portable air filters in a classroom environment. However, these studies investigating the potential benefit of portable air cleaners with HEPA filters in classrooms have not measured infiltration of UFPs [48–57]. Yang et al. conducted a double-blind crossover study investigating the pulmonary benefits of a HEPA air purifier intervention in 125 school children in China [48]. The study found that the intervention was associated with a decrease in runny nose, FeNO, and markers of systemic inflammation [48].

A randomized crossover study of HEPA filtration, without a washout period, in 23 homes of low-income Puerto Ricans in Boston and Chelsea, MA, concluded that a portable HEPA filter intervention significantly improved of indoor air quality [58]. Median UFP concentration when using HEPA filtration was 50% to 85% lower compared to no filtration in most homes.

Existing literature supports the notion that in-class performance of students is directly impacted by the air pollution level at their school. In Los Angeles, researchers studied how changes in ambient air pollution concentrations affected the performance of second- through sixth-grade students on standardized tests between 2002 and 2008 [59]. Comparisons were made between different cohorts within the same school to account for differences between schools, including differences in outdoor pollution, socioeconomic status of students and other factors that vary between schools. Researchers found that lower concentrations of daily outdoor particulate matter significantly increased mathematics and reading test scores. Similar associations between test scores and short-term air pollution concentrations have been observed nationally and internationally.

The findings of Phase 1 of the Healthy Air, Healthy Schools Project are consistent with existing literature demonstrating that HEPA filter interventions reduce exposure to outdoor pollutants in indoor spaces. This study is unique in focusing on UFPs in school settings and demonstrating through multivariate methods that the UFPs measured in the classroom space are primarily of outdoor origin. Although existing research suggests that improvements to indoor air quality in homes can significantly improve asthma outcomes, further investigation is necessary to establish the benefits to student health and academic performance of improved air quality in schools.

## 5. Conclusions

Indoor air quality in schools is significantly impacted by outdoor sources of UFPs. Portable HEPA filters can substantially reduce the concentration of outdoor pollution in the classroom. Using portable HEPA filter units reduced indoor concentrations of UFPs by approximately 70%. Schools that are near truck routes, aircraft flight paths, and high-traffic roadways are at higher risk of indoor air pollution. Landing aircraft contribute significantly to indoor and outdoor UFP concentrations in this study region. Portable HEPA filter units can be effectively used in the short term to decrease air pollution in classrooms by removing particles. Ventilation changes and building-level remediations such as sealing gaps and managing doorways should be investigated as an approach to reduce infiltration of outdoor particles indoors. The next phase of the project will evaluate the (1) optimal usage of HEPA filter units to balance energy usage and air quality management, (2) health and well-being benefits of reduced UFP concentrations indoors, and (3) methodologies to identify schools at higher risk UFP impacts.

## Supplementary Material

Supplement

## Figures and Tables

**Table 1.** School classroom dimension and volume.

School	Distance from Airport	Room Area, Full Dimensions (ft) †	Ceiling Height (ft)	Room Volume (ft^3^)	Room Volume (m^3^)
**School A 1st flr.**	1.5 miles	30.2 × 29.2	9.9	8725.3	247.1
**School A 2nd flr.**	1.5 miles	35.0 × 28.0	9.9	9227.5	261.3
**School B**	2.1 miles	32.0 × 28.7	9.3 to 12.9 ‡	10,053.7	284.7
**School C**	7.2 miles	31.8 × 26.5	10.4	8750.3	247.8
**School D**	0.5 miles	31.7 × 23.1	9.1	6574.7	186.2
**School E**	5.3 miles	32.0 × 30.0	8.2	7840	222.0

† Some classrooms have walled-off corner sections so are not fully rectangular.

‡ Sloping ceiling, minimum height near windows and maximum by interior hallway.

**Table 2.** Air quality instruments used to measure conditions in classroom and ambient air.

Parameter	Instrument	Manufacturer	Averaging Time
CO_2_	LI-850 CO_2_	Li-Cor Biosciences	10 s
Ultra-fine particle size distribution	NanoScan	TSI, Inc.	1 min (full scan)
Particles > 10 nm count	CPC	TSI, Inc.	10 s
Particles > 20 nm count	P-Trak	TSI, Inc.	10 s
Black carbon	MA200	AethLabs	10 s
Black carbon	AE51	AethLabs	10 s
Temperature, RH	Hobo sensor	Onset Computer Corp.	10 s

**Table 3.** School classroom visit dates and aircraft operations at school location.

School and Room	First Visit	Sea-Tac Flight Operations	Second Visit	Sea-Tac Flight Operations
School A 1st flr.	June 9–11	Landing	July 26–28	Take off
School A 2nd flr.	June 14–16	Landing 14th and 15th Take off 16th	July 28–30	Take off
School B	April 14–16	Take off	July 20–22	Landing 20th and 21st Take off 22nd
School C	April 7–9	Take off	July 13–15	Take off
School D	June 22–24	Take off	August 10–12	Landing
School E	March 24–26	Take off 24th and 26th Landing 25th	July 7–9	Take off

**Table 4.** Percentage of missing or error flagged data.

Location	Percent Instrument Error (%)
School A Classroom 1, Visit 1	0
School A Classroom 1, Visit 2	43
School A Classroom 2, Visit 1	0
School A Classroom 2, Visit 2	33
School B Visit 1	0
School B Visit 2	27
School C Visit 1	0
School C Visit 2	0
School D Visit 1	0
School D Visit 2	0
School E Visit 1	19
School E Visit 2	0

**Table 5.** Outdoor air exchange rate (AER Outdoor).

School	AER Visit 1	AER Visit 2
School A		
Room #1	2.1/h	1.3/h
Room #2	4.4/h	1.1/h
School B	0.6/h	0.9/h
School C	2.2/h	2.6/h
School D	2.9/h	0.4/h
School E	1.1/h	1.1/h

**Table 6.** Infiltration (%) with and without the portable HEPA filter unit.

Pollutant Type	Infiltration before HEPA	Confidence Range (%)	Infiltration after HEPA	Confidence Range (%)	Removal by HEPA (%)	Confidence Range
Total UFP	54%	47–59	9%	8–9	83%	82–84
Aircraft Particles	41%	38–56	14%	12–15	67%	67–73
Black Carbon	74%	71–79	20%	18–21	73%	73–74

**Table 7.** Number of arrivals and departures below 750 m and within a one-mile radius of King County Schools in 2019. Flight information including the median and 25th–75th percentiles are provided for the three airports in the region.

Airport	# Arrivals		# Departures	
	Median	(25th–75th Percentile)	Median	(25th–75th Percentile)
**Sea-Tac Airport**	3	(0–36)	10	(3–24.75)
**Boeing Field**	44.5	(7–232)	11	(0–60)
**Renton Municipal Airport**	0	(0–1)	0	(0–0)
**All Airports**	102.5	(18–589)	35	(5204.75)

**Table 8.** Number of arrivals and departures below 750 m and within a one-mile radius of study schools in 2019. Flight information including the median and 25th–75th percentiles are provided for the three airports in the region.

Airport	# Arrivals		# Departures	
	Median	(25th–75th Percentile)	Median	(25th–75th Percentile)
**Sea-Tac Airport**	61,234	(59,529–154,962)	53,313	(6157–55,589)
**Boeing Field**	18	(8–89)	26	(25–104)
**Renton Municipal Airport**	0	(0–0)	0	(0–0)
**All Airports**	61,240	(59,537–155,094)	53,547	(6183–55,693)

## Data Availability

All data produced in the present study are available upon reasonable request from the authors.

## Abbreviations

AER: Air exchange rate
AHAM: Association of Home Appliance Manufacturers
AQS: Air Quality System
BC: Black carbon
BFI: Boeing Field
CADR: Clean air delivery rate
CFM: Cubic feet per minute
CPC: Condensation particle counter
FeNO: Fractional exhaled nitric oxide
HEPA: High-efficiency particulate air filter
MOV-UP: Mobile Observations of Ultrafine Particles
OSPI: Office of Superintendent of Public Instruction
RNT: Renton Municipal Airport
Sea-Tac: Seattle-Tacoma International Airport
SES: Socioeconomic
UFPs: Ultrafine particles
Ultra-UFPs: Ultra-ultrafine particles
