# Supplementary material for: Indoor Air Quality Intervention in Schools: Effectiveness of a Portable HEPA Filter Deployment in Five Schools Impacted by Roadway and Aircraft Pollution Sources
Source: Atmosphere (Basel). Author manuscript; Available in PMC 2024 Aug 29. (PMC11361409; doi:10.3390/atmos13101623)
Supplement: Supplement [file NIHMS1962880-supplement-Supplement.pdf]

Indoor Air Quality Intervention in Schools: Effectiveness of a Portable HEPA Filter Deployment in Five Schools Impacted by Roadway and Aircraft Pollution Sources

Nancy Carmona [1,*], Edmund Seto [1], Timothy R. Gould [2], Everetta Rasyid [1], Jeffry H. Shirai [1], BJ Cummings [1], Lisa Hayward [1], Timothy V. Larson [1,2] and Elena Austin [1]

[1] Department of Environmental & Occupational Health Sciences, University of Washington, WA 98195,
[2] Department of Civil & Environmental Engineering, University of Washington, WA 98195, USA
* Correspondence: nancyc9@uw.edu

We plotted timeseries data of the indoor to outdoor total particle count for visits which had valid NanoScan data. Several visits resulted in insufficient valid NanoScan data for School A – 1st floor Visit 2, School A – 2nd floor Visit 2, School B Visit 1, and School E Visit 1.

Figure S1. Plots of the ratio of indoor to outdoor total particle count concentration at all school visits with valid NanoScan data.

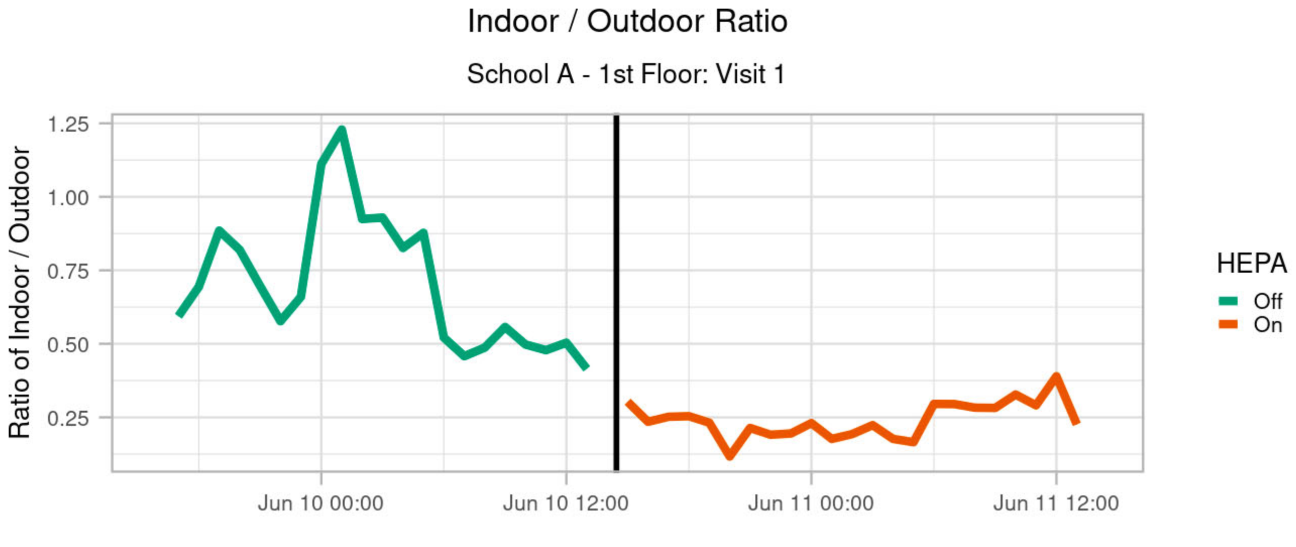

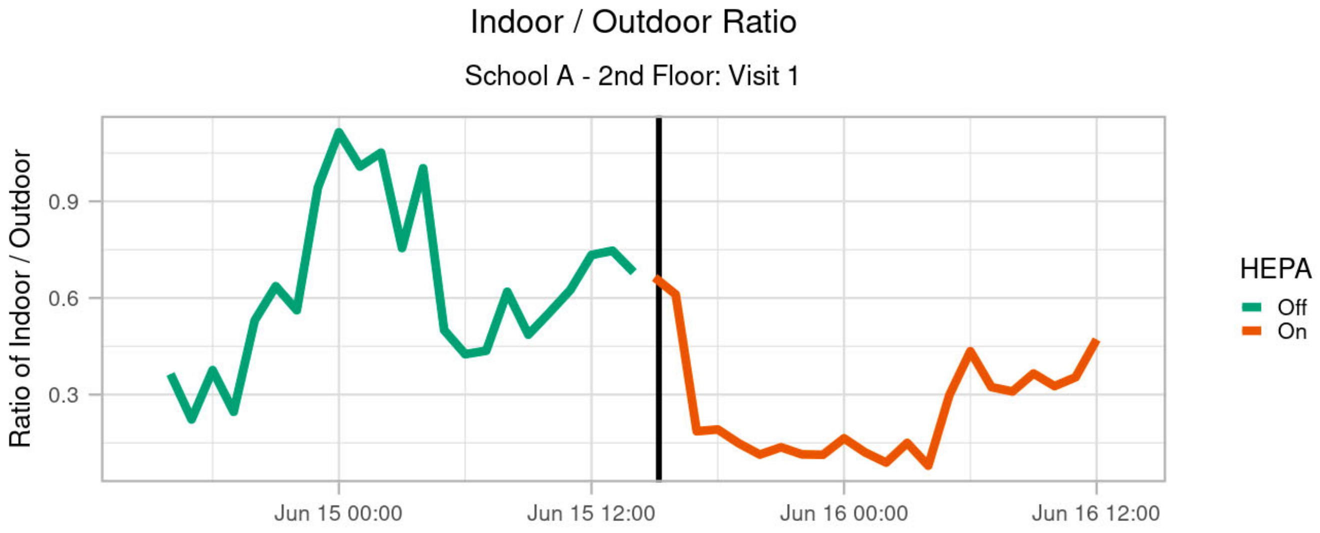
Indoor / Outdoor Ratio
School A - 2nd Floor: Visit 1
Ratio of Indoor / Outdoor
0.3
0.6
0.9
Jun 15 00:00
Jun 15 12:00
Jun 16 00:00
Jun 16 12:00
HEPA
Off
On

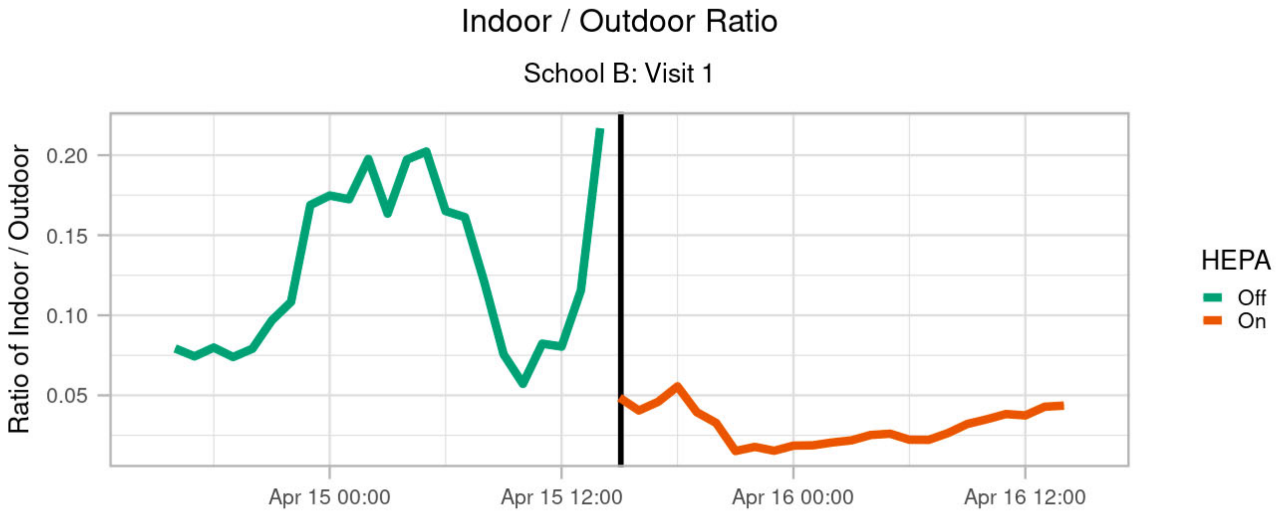
Indoor / Outdoor Ratio
School B: Visit 1
Ratio of Indoor / Outdoor
0.05
0.10
0.15
0.20
Apr 15 00:00
Apr 15 12:00
Apr 16 00:00
Apr 16 12:00
HEPA
Off
On

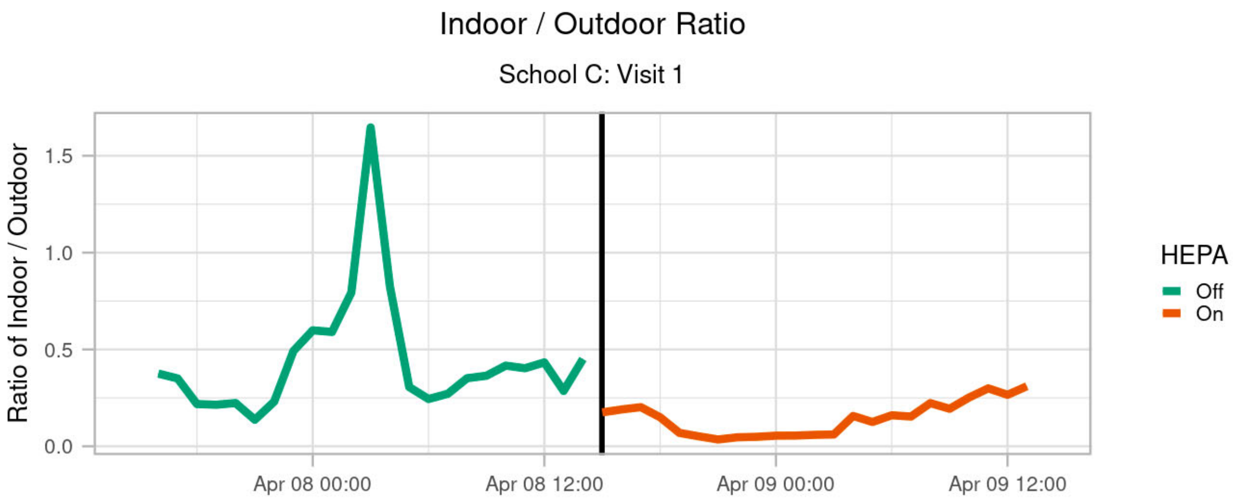
Indoor / Outdoor Ratio
School C: Visit 1
Ratio of Indoor / Outdoor
1.5
1.0
0.5
0.0
Apr 08 00:00
Apr 08 12:00
Apr 09 00:00
Apr 09 12:00
HEPA
Off
On

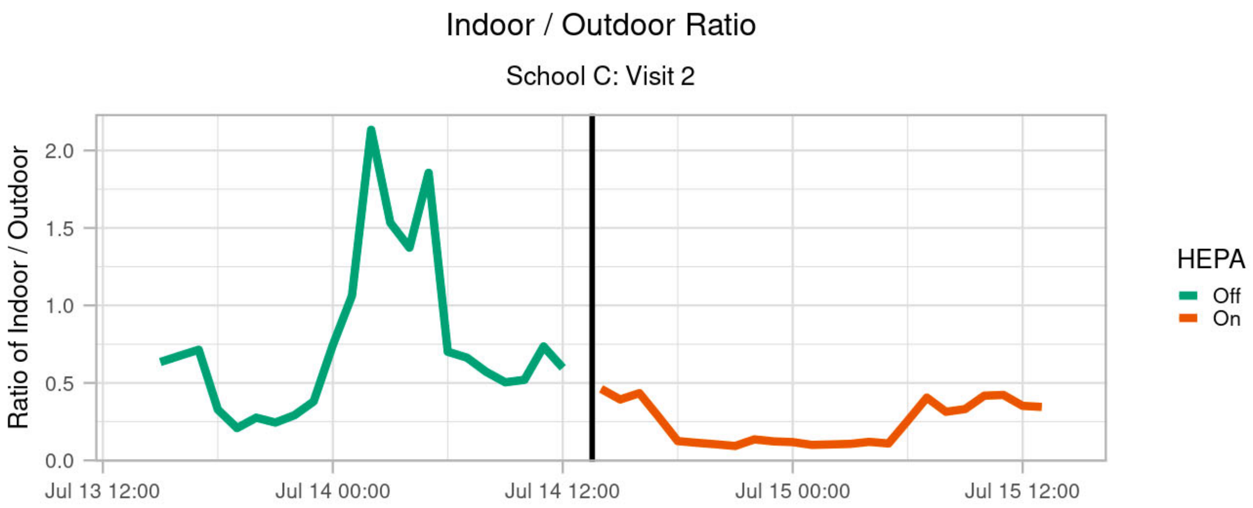
Indoor / Outdoor Ratio
School C: Visit 2
Ratio of Indoor / Outdoor
2.0
1.5
1.0
0.5
0.0
Jul 13 12:00
Jul 14 00:00
Jul 14 12:00
Jul 15 00:00
Jul 15 12:00
HEPA
Off
On

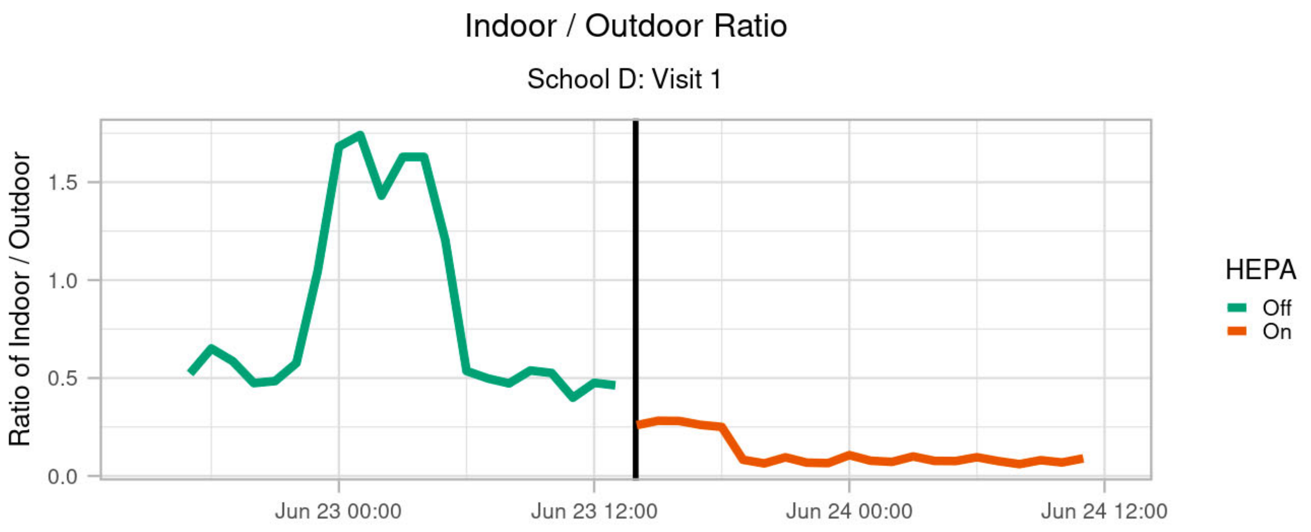
Indoor / Outdoor Ratio
School D: Visit 1
Ratio of Indoor / Outdoor
0.0
0.5
1.0
1.5
Jun 23 00:00
Jun 23 12:00
Jun 24 00:00
Jun 24 12:00
HEPA
Off
On

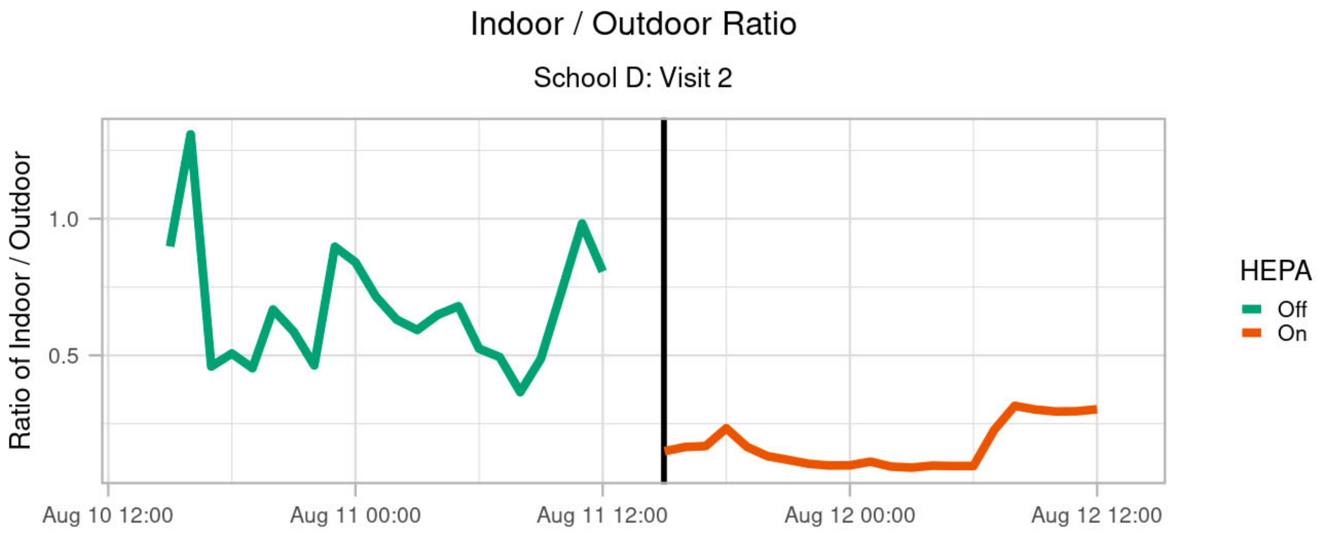
Indoor / Outdoor Ratio
School D: Visit 2
Ratio of Indoor / Outdoor
0.5
1.0
Aug 10 12:00
Aug 11 00:00
Aug 11 12:00
Aug 12 00:00
Aug 12 12:00
HEPA
Off
On

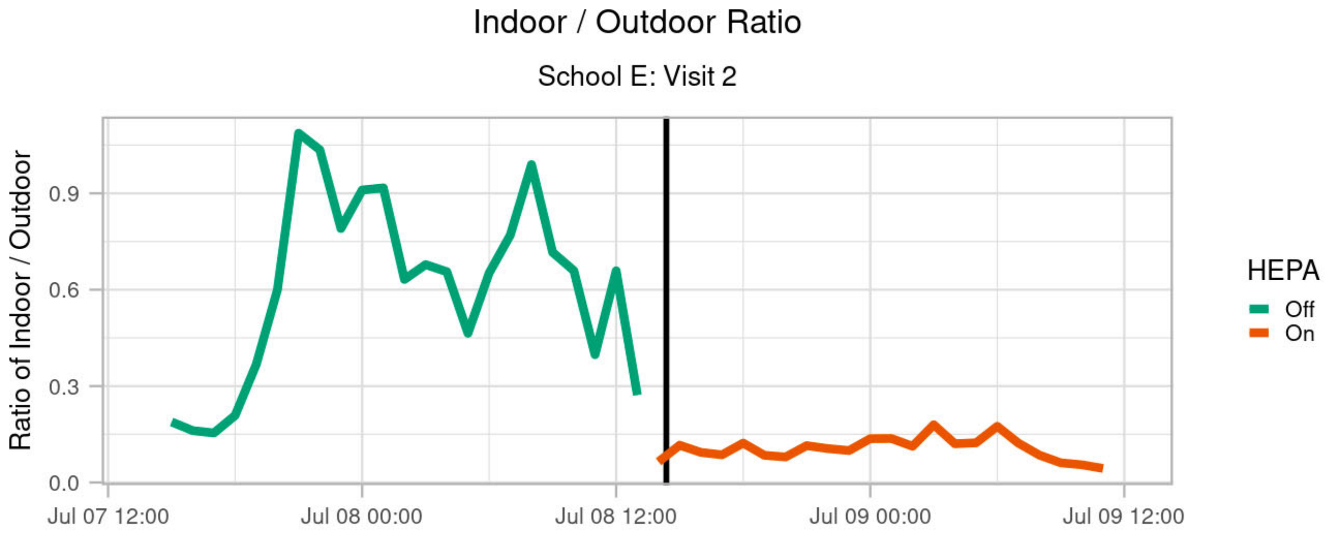
Indoor / Outdoor Ratio
School E: Visit 2
Ratio of Indoor / Outdoor
0.9
0.6
0.3
0.0
Jul 07 12:00
Jul 08 00:00
Jul 08 12:00
Jul 09 00:00
Jul 09 12:00
HEPA
Off
On
